# The Removal of an Inverted Womb

**Published:** 1871-12

**Authors:** 


THE REMOVAL OF AN INVERTED WOMB.—Dr. Thomas Hay, of York, Penn. (*Med. and Surg. Reporter*), lately removed an inverted uterus with an intramural fibrous tumor of the fundus. Four weeks afterward the patient was up and about her room, and the operation bade fair to be a perfect success.—*N. Y. Med. Record.*

